# Physiological and pathological roles of Ang II and Ang- (1-7) in the female reproductive system

**DOI:** 10.3389/fendo.2022.1080285

**Published:** 2022-12-23

**Authors:** Yuanyuan Liu, Haomeng Hao, Tingting Lan, Rui Jia, Mingya Cao, Liang Zhou, Zhiming Zhao, Wensen Pan

**Affiliations:** ^1^ Department of Reproductive Medicine, The Second Hospital of Hebei Medical University, Shijiazhuang, China; ^2^ Shenzhen Key Laboratory of Reproductive Immunology for Peri-Implantation, Shenzhen Zhongshan Institute for Reproduction and Genetics, Shenzhen Zhongshan Urology Hospital, Shenzhen, Guangdong, China; ^3^ Second Department of Respiratory and Critical Care Medicine, The Second Hospital of Hebei Medical University, Shijiazhuang, Hebei, China

**Keywords:** Angiotensin II, Angiotensin-(1-7), Renin-Angiotensin System, ovary, endometrial, placenta

## Abstract

The local Renin-Angiotensin System (RAS) has been demonstrated to exist in a wide range of tissues and organs, In the female reproductive system, it is mainly found in the ovary, uterus and placenta. The RAS system is made up of a series of active substances and enzymes, in addition to the circulating endocrine renin-angiotensin system. The active peptides Angiotensin II (Ang II) and Angiotensin (1-7) (Ang-(1-7)), in particular, appear to have distinct activities in the local RAS system, which also controls blood pressure and electrolytes. Therefore, in addition to these features, angiotensin and its receptors in the reproductive system seemingly get involved in reproductive processes, such as follicle growth and development, as well as physiological functions of the placenta and uterus. In addition, changes in local RAS components may induce reproductive diseases as well as pathological states such as cancer. In most tissues, Ang II and Ang- (1-7) seem to maintain antagonistic effects, but this conclusion is not always true in the reproductive system, where they play similar functions in some physiological and pathological roles. This review investigated how Ang II, Ang- (1-7) and their receptors were expressed, localized, and active in the female reproductive system. This review also summarized their effects on follicle development, uterine and placental physiological functions. The changes of local RAS components in a series of reproductive system diseases including infertility related diseases and cancer and their influence on the occurrence and development of diseases were elucidated. This article reviews the physiological and pathological roles of Ang II and Ang- (1-7) in female reproductive system,a very intricate system of tissue factors that operate as agonists and antagonists was found. Besides, the development of novel therapeutic strategies targeting components of this system may be a research direction in future.

## Introduction

It is commonly known that the ovary, uterus, and placenta all contain local renin-angiotensin systems (1). However, its physiological and pathological significance in female reproduction is still unknown. Ang II and Ang- (1-7) are two of the RAS system’s prominent active peptides. Ang II and Ang- (1-7) are bioactive substances involved in blood pressure regulation. These compounds are mostly linked to the incidence and progression of cardiovascular disorders (2). The biological effects of Ang-(1-7) are often in opposition to those of Ang II (3). With the depth of its research, recent studies have shown that it has a close bearing on physiological functions and pathological changes of the reproductive system (4–6).

The AT1 and AT2 receptors are the two main subtypes of Ang II in classical RAS, which is generated by Ang I in response to ACE (7). Ang II acts through AT1 using various signaling mechanisms, similar in some ways to those brought on by the EGF family and hormones mobilized by Ca 2+. On the contrary, phosphatases are primarily used to convey signals caused by activation of the AT 2 receptor. As a result, AT 2 receptor activation is thought to inhibit AT 1 receptor-mediated signaling (8).

**Figure 1:**
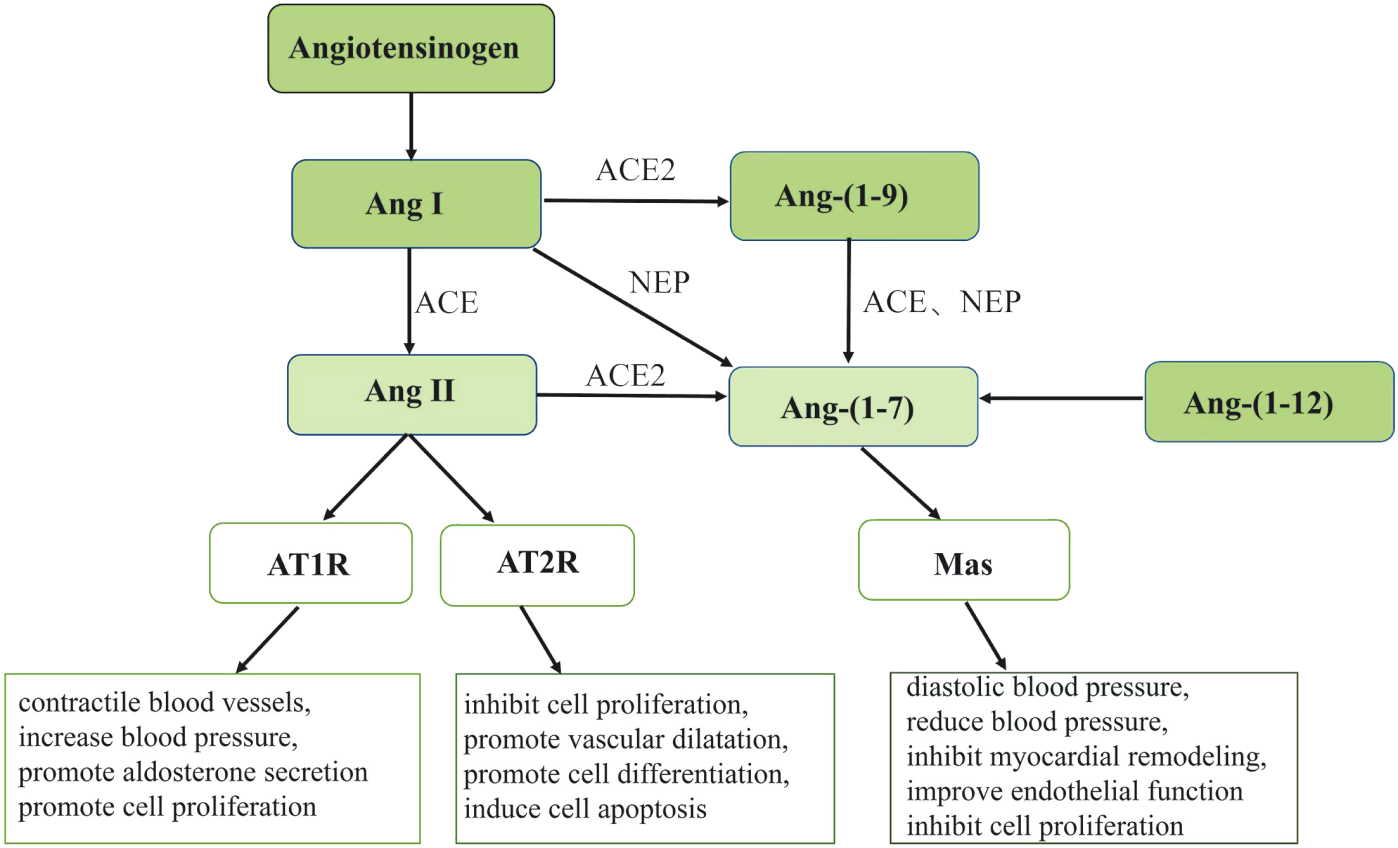
Ang, angiotensin; ACE, angiotensin converting enzyme; ACE2,angiotensin converting enzyme 2; AT1R, angiotensin receptor 1; AT2R, angiotensin receptor 2; NEP, enkephalin enzymes. The figure shows the pathway of Ang II and Ang- (1-7) generation. Angiotensinogen can generate Ang I and then hydrolyze Ang I to Ang II under the action of ACE. The generated Ang II can play different physiological roles through different receptors AT1R and AT2R.Ang- (1-7) can be generated through four pathways under the action of different enzymes and exert its physiological effects through Mas receptors.

Ang-(1-7) is generated through four metabolic pathways. The first is the hydrolysis of Ang I by enzymes such as enkephalin (NEP). Secondly, the angiotensin converting enzyme 2 (ACE2) cleaves Ang II to produce Ang-(1-7). Thirdly, ACE and NEP hydrolyze Ang-(1-9) to form Ang-(1-7). Additionally, Ang- (1-7) can be produced directly from Ang- (1-12) (9, 10). Among them, the cleavage of Ang II by ACE2 is the primary mechanism of production of Ang- (1-7) (11, 12). Although previous literature has demonstrated that Ang- (1-7) can act through AT1, AT2 and MAS receptors, it has radically differing affinities for AT1 and AT2 receptors (13, 14). MAS1 proto-oncogene G protein-coupled receptors are another route *via* which Ang- (1-7) can exert its effects (15), this is the main way that Ang- (1-7) plays its function. Ang II can contractile blood vessels, increase blood pressure, promote aldosterone secretion and promote cell proliferation through AT1 receptor. Ang II can inhibit cell proliferation, promote vascular dilatation, promote cell differentiation and induce cell apoptosis through AT2 receptor. Ang- (1-7) can diastolic blood vessels, reduce blood pressure, inhibit myocardial remodeling, improve endothelial function and inhibit cell proliferation through Mas receptor (**Figure 1**).

Changes in local Ang II and Ang- (1-7) activity can cause illnesses of the reproductive system, which in turn can lead to fertility issues. The content of this review is to describe the positive and negative effects of Ang II, Ang-(1-7) and their receptors on the biology of the ovary, the uterus, and the placenta. It provided an overview of Ang II and Ang- (1-7) expression, location, metabolism, and action in many reproductive organs, and elucidated their roles in physiological functions of the reproductive system. Finally, the variations in Ang II and Ang- (1-7) expression and activity in pertinent disorders were investigated. Additionally, their effects on the onset and progression of illnesses as well as their potential for the therapy of connected disorders were explored.

## Ang II, Ang- (1-7) and physiology of female reproductive system ovary

The primary human organ from which plasma reninogen is derived is the ovary (12).

Renin, which catalyzes the transformation of angiotensinogen into Ang I, is a precursor of prorenin. Ang I can either be immediately hydrolyzed to Ang- (1–7) or transformed into Ang II. Following that, Ang II was hydrolyzed by angiotensin converting enzyme 2 (ACE2) (16).

The follicular fluid contains Ang II and Ang- (1-7). While Ang- (1-7) is substantially less concentrated in the follicular fluid than it is in the plasma, Ang II is more abundant in the follicular fluid than it is in the plasma (2). This difference may be owing to the extra-ovarian origin of Ang II or the shorter half-life of Ang- (1-7) in follicular fluid. There was evidence that AT1R and AT2R were present in granulosa cells of primordial, primary, and secondary follicles (17). In the corpus luteum, microvascular endothelial cells express both AT1R and AT2R, although their expression varies during the course of the cycle: While AT1R expression does not change, AT2R expression decreases in the middle of the luteal phase and increases in the late luteal phase (14–16). All developmental stages of follicles are immune-responsive to Ang-(1-7) and Mas, except for the corpus luteum, ovarian stroma, and blood vessels (2, 18). The expression of Ang- (1-7) and Mas also varies in different developmental stages of follicles. In the granulosa layer of follicles, immunostaining can be observed in primary follicles, and strong immune response can be observed in secondary follicles. The Ang- (1-7) labeling is weak in preovulatory follicles, and Mas is moderately strong labeling. In secondary follicles, there was a significant immunoreactivity for Ang- (1-7) and Mas, and both were negative in preovulatory follicles. In the cytoplasm of large and small luteal cells, Ang- (1-7) labeling was weak, but Mas labeling was strong. Postmenopausal women’s ovarian stromal cells stained moderately with Ang- (1-7) and Mas (2, 18).

Relevant studies have demonstrated that Ang II and its receptors have regulatory effects on oocyte maturation (19–22), *In-vitro* culture of bovine cumulus oocyte complex with Ang II can induce oocyte nucleus maturation (21, 23). Aside from that, Ang II also contributes to ovulation. Ang II can affect ovulation in cattle and rats through AT2R. Moreover, this effect can be prevented by inhibiting AT2R (24). Furthermore, the results showed that AT2R was also involved in follicular atresia through apoptosis (25, 26). Also, Ang- (1-7) participates in oocyte maturation and ovulation. Ang- (1-7) can promote the recovery of oocyte meiosis and induce ovulation in the absence of gonadotrophins, and an antagonist specific to Ang-(1-7), A779, inhibits these functions (18).

The plasma Ang-(1-7) levels in gonadotropin-stimulated women were shown to be elevated in a study of patients undergoing ovarian stimulation (COS) with human urine gonadotropin (HMG) and/or recombinant FSH (rFSH) (18) (2). Additionally, a linear relationship between Ang-(1-7) concentrations in human follicular fluid and the percentage of developed oocytes was discovered. However, in women with poor ovarian reserve function, AT1 receptor expression in granulosa cells was inversely linked with the amount of mature oocytes (27) (**Table 1**)

**Table 1:** Expression status of RAS components in different parts of ovary.

Ovarian structures	Ang II	Ang-(1-7)	AT1R	AT2R	Mas
Blood vessel	+	–	+	+	–
Primordial follicles		+		+	+
Oocytes	+		+		
Pregranulosa cells	+		–		
Primary/intermediate follicles		+		+	+
Oocytes	+		+		
Granulosa cells	+		+		
Secondary/Graafian follicles		+		+	+
Oocytes	+		+		
Granulosa cells	+		+		
Theca cells	+		+		
Ovarian cortex				n	
Epithelial cells	+	n	–		n
Stromal cells	+	+	+		+
Corpus luteum (early luteal phase)					
Large luteal cells	+	+	+	+	+
Small luteal cells	+	+	+	+	+
Corpus luteum (midluteal luteal phase)					
Large luteal cells	+	+	+	+	+
Small luteal cells	+	+	+	+	+
Corpus luteum (late luteal phase)					
Large luteal cells	+	+	+	+	+
Small luteal cells	+	+	+	+	+
Corpus luteum (in pregnancy)		n		n	n
Large luteal cells	+		+		
Small luteal cells	+		+		

+: express;-: negative; n: no data.

The expression and distribution of Ang II, Ang- (1-7) and their receptors are not the same among different species in ovary, which indicates that Ang II, Ang- (1-7) and their receptors may have different physiological effects. As the main bioactive substances in the renin angiotensin system, Ang II and Ang- (1-7) generally perform different functions and maintain a relative balance. Interestingly, in the ovary both seem to act in the same direction and play a crucial part in the physiological function of the ovary.

## Endometrial

**Figure 2:**
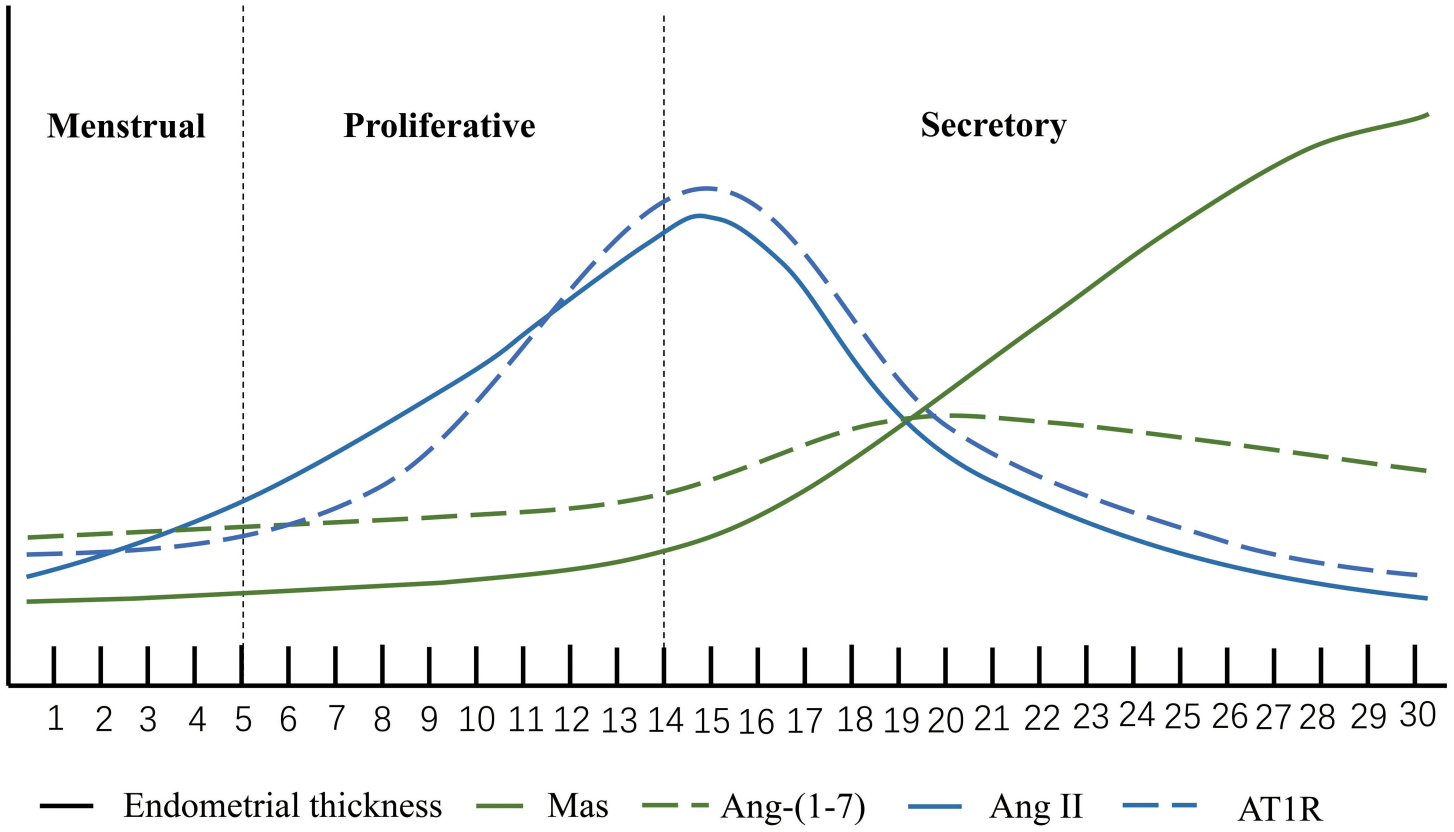
Ang II, Ang-(1-7), AT1R and Mas. The horizontal axis represents the menstrual cycle. The height of the vertical coordinate. The expression level of the molecule at each stage of the menstrual cycle.

The endometrium undergoes periodic proliferation, differentiation and shedding during the menstrual cycle. Changes in the endometrium, driven by ovarian steroid hormones, play a crucial role in embryo implantation and pregnancy establishment (28). As reported by previous studies, Ang II and Ang- (1-7) and their related receptors can be detected in the endometrium throughout the menstrual cycle and change with the menstrual cycle (29). Ang II is most expressed in the proliferative phase, when it is predominantly located in glandular epithelium and stroma (30). Its expression is reduced during the secretory phase and is restricted to vascular stromal cells surrounding spiral arterioles in the endometrium (30, 31). The uterus has expression of both the Ang II receptor type 1 (AT1) and the Ang II receptor type 2 (AT2) proteins. The majority of AT1 is found in the endometrium, which has a high level of expression during the late proliferative phase but a lower level of expression during the secretory phase. AT2 was principally expressed in myometrium and uterine artery (32). Some studies have suggested that Ang II receptor activation can bring about apoptotic changes in the vascular wall (33). therefore, Ang II may be involved in endometrial vasoconstriction as well as vascular remodeling during menstruation (34). The expression of Ang- (1-7) was weak in endometrial glands at the early and middle stages of proliferation. Nonetheless, at the late stage of secretion, the expression of Ang- (1-7) increased and peaked. Ang- (1-7) was detected in the early stage of endometrial stromal hyperplasia, but not in the endothelium of endometrial vessels in the late secretory stage (30). The Ang- (1-7) receptor Mas, in contrast to Ang II receptor, only minimally enhanced in the glandular epithelium of the middle and late secretory stage, and did not change significantly with the fluctuation of ovarian steroid hormones. An experiment in which ovariectomized rats were given with estrogen or estrogen in combination with progesterone provided additional evidence that the expression of Mas receptors in the endometrium is not regulated by female sexual hormones (35, 36) (**Figure 2**)

However, there are still few studies bound up with the physiological function of local renin-angiotensin system in the uterus. Two studies on human endometrial cells cultured *in vitro* found that Ang II could induce cell proliferation and increase cell activity, but these effects could be offset by adding Ang- (1-7) (37, 38). Ang II can promote the transdifferentiation of endometrial epithelial cells into myofibroblasts. Except for this, Ang II has the ability to induce the activation of collagen type I as well as fibronectin. A recent study significantly prevented the biochemical and histopathologic changes resulted from endometrial hyperplasia by treating rats with angiotensin II receptor blockers. This means that the imbalance of local uterine RAS system and Ang II activation may trigger endometrial fibrosis, but this effect can also be inhibited by Ang- (1-7) (39, 40).

## Placenta

**Figure 3:**
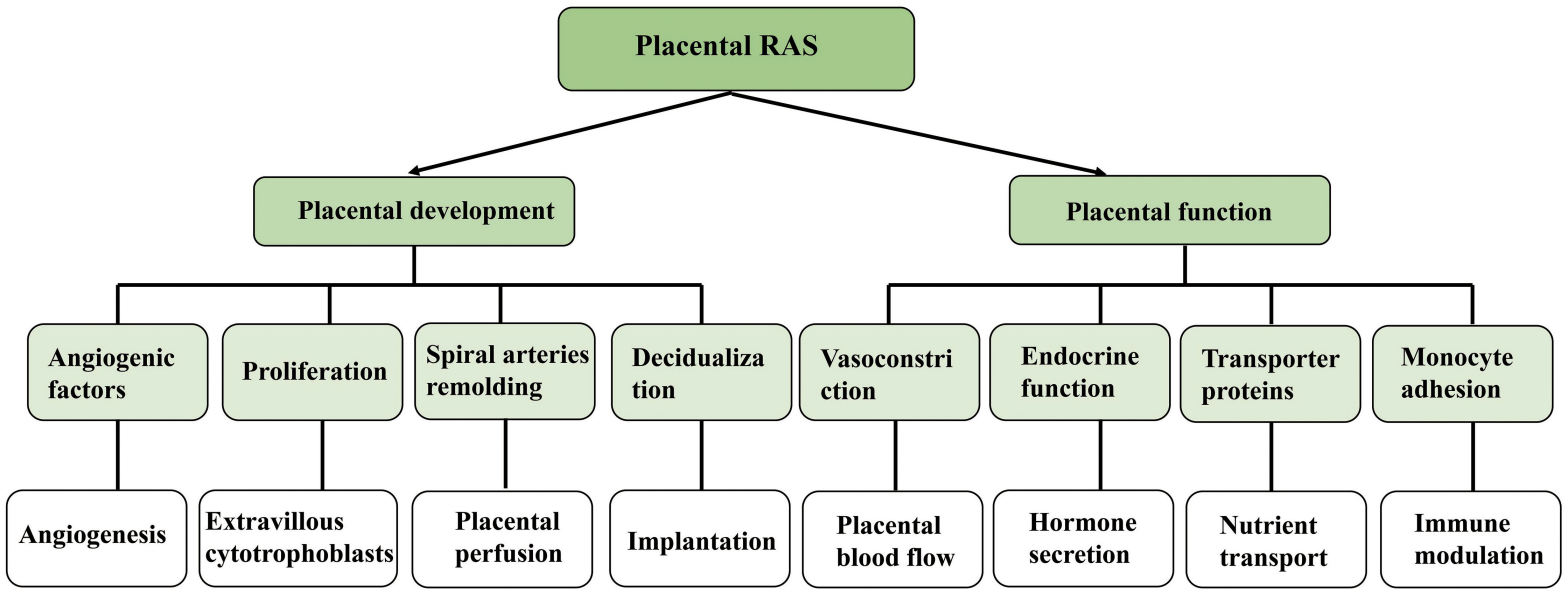
Effect and function of local renin angiotensin system on embryonic development.

As a specialized connection for maternal exchange, the placenta supports hormone secretion, gas and nutrient exchange, as well as the establishment of an immune barrier (41). In the placental villi, high quantities of Ang II were found, and the expression of renin and ACE was also observed. AT1R and AT2R were detected in trophoblast cells (42). Different from the weak expression of AT2R, the expression of AT1R was quite high at the transcriptional level as well as the translational level throughout the whole pregnancy (42). All these circumstances suggest that placenta also has local RAS. As early as the second week of embryonic development, local RAS can promote blastocyst implantation, and Ang II can also heighten endometrial cell permeability by promoting decidual cell differentiation, thereby allowing trophoblast cell invasion into the maternal endometrium (43). Numerous studies have shown the pivotal function of uteroplacental RAS in spiral arterial remodeling and angiogenesis (44, 45). It has been shown that Ang II can influence the production of vascular endothelial growth factor (VEGF) and placental growth factor (PlGF) in vascular endothelium and smooth muscle cells *via* activating AT1R in these cell types (46). In normal pregnancy, both VEGF and PlGF may be found in the trophoblast as well as the maternal decidua. In addition to this, VEGF and PlGF play a significant part in the development of the placental vasculature by controlling the growth of blood vessels in the chorionic villi (47). AT1R signaling in trophoblast cells also helps to enhance the synthesis of antiangiogenic molecules such as soluble Flt1 (sFlt1) and soluble endocrine hormone (sEng) (48), which helps to maintain a healthy equilibrium in placental angiogenesis. In addition to upregulation that occurs in trophoblast as a result of AT1R activation, sFlt1 expression also activates AT2R and Mas receptors *via* the binding of Ang II and Ang (1-7), respectively. This is not the case with sEng, indicating that the control of sEng is primarily dependent upon AT1R signaling and does not call for the involvement of AT2R or Mas receptors (49–51) (**Figure 3**)

It has been shown that angiotensin II is involved in the control of placental blood flow, hormone production, and immunological function. According to one research, Ang II may cause vasoconstriction in separate chorionic plate arteries (52). Pregnant mice lacking AT2R experience a gradual rise in blood pressure, indicating that AT2R signaling *in utero*placental arteries is crucial for maintaining a healthy placental perfusion balance (53, 54). Ang II may modify the impact of nutrition transport by affecting blood vessels. The enhanced secretion of hPL and SP1 and the synthesis of estrogen might both be boosted by Ang II, according to *in-vitro* tests on placental explants (48). The use of certain pharmacological blockers lends credence to the hypothesis that this function is mediated by AT1R and is associated with calcium signaling. In addition, it has been shown that Ang II, when co-cultured with trophoblast cells, affects monocyte adhesion *in vitro* and modulates immunity in the placenta (55–57).

There are few studies on the expression, localization and function of Ang- (1-7) in the placenta, and more evidence is needed to reveal its interaction with the placenta.

## Ang II, Ang- (1-7) and the pathophysiology of female reproductive system ovarian hyperstimulation syndrome

The most dangerous side effect of ovulation induction is a condition called ovarian hyperstimulation syndrome (OHSS), which has a high fatality rate as well as a high morbidity rate. Ovarian enlargement, increased vascular permeability, extracellular fluid accumulation, and electrolyte imbalance are the hallmarks of severe OHSS (58).

High renin activity was found in both follicular fluid and plasma in patients with OHSS and was found to be directly associated with the severity of ovarian hyperstimulation syndrome (59–61). Several results showed that ascites from patients with severe OHSS had considerably greater amounts of reninogen and angiotensin II than pleural effusion and plasma did (62, 63). This result suggests that besides the peripheral circulation, the RAS system is also activated locally in the ovary. Activated ovarian RAS may cause ovarian enlargement and extracellular fluid accumulation in OHSS patients by inducing neovascularization and increasing capillary permeability.

Several researches have evaluated the effectiveness of medications that inhibit angiotensin converting enzyme and drugs that block angiotensin II receptors (64). Enalapril oral treatment was thought to have a 40% reduction in the occurrence of OHSS in a rabbit model, according to Morris et al. Nonetheless, Sahin et al. observed that neither hindering ACE nor blocking AngII receptor improved the ascites of OHSS in rabbits (65). Two clinical trials investigated the efficacy of dual renin-angiotensin blockade (a combination of angiotensin receptor blocker and angiotensin converting enzyme inhibitor) in preventing OHSS in IVF patients. It was found that this treatment may prevent some of the symptoms of OHSS and maintain a higher pregnancy rate after transfer of frozen-thawed embryos. However, OHSS cannot be completely eliminated by this treatment (66, 67).

## Polycystic ovary syndrome

Polycystic ovary syndrome (PCOS) is a complex illness that affects both the endocrine and metabolic systems. Its typical clinical phenotype includes Hyperandrogenism, insulin resistance, anovulation/anovulation and polycystic changes of the ovary are the most common causes of anovulation infertility (68).

There have been some studies on the expression changes of RAS components in patients with PCOS (69). According to Alphan et al., PCOS patients had greater blood total renin concentrations than non-PCOS patients (70). According to a survey, total renin concentrations were greater in obese PCOS women, and they were associated with fasting insulin and free testosterone levels. Although there was no discernible difference between PCOS women and controls in terms of plasma renin activity, ACE activity and Ang II levels were both markedly elevated in PCOS patients (71). Based on these findings, PCOS patients have an active renin-angiotensin system. A recent research, however, found that the ACE2-Ang- (1-7) -Mas axis in the ovary was hindered in polycystic ovarian rats. But in this study, a rat model characterized only by polycystic ovaries was used. Under the circumstances, RAS system expression levels are affected by multiple hormonal changes. As a consequence, the results of this experiment cannot preferably express the changes of RAS components in PCOS (72). In the endometrium of PCOS patients, a study of 15 endometrium of PCOS patients showed that the mRNA expression levels of ACE2, AT1, AT2 and Mas receptors in the endometrium of PCOS patients were higher than those in the control groups (73), These results make it clear that the elevated expression level of RAS components may affect the development of endometrium and play a role in its pathological process.

Insulin resistance and hyperandrogenemia are important clinical expressions of PCOS, and they are not independent. For example, insulin resistance can induce the increase of androgen level. As a multitude of studies have shown that Ang- (1-7) receptors can alleviate a myriad of metabolic diseases through Mas receptors and also counteract a range of adverse effects of Ang II, hypertensive drugs that block the action of Ang II can improve insulin resistance (74). Insulin resistance and hyperandrogenemia are the most common symptoms in PCOS patients. Studies have shown that Ang- (1-7) can not only improve the action of insulin, but also increase the delivery of insulin to target organs through vasodilation (75). A range of evidence confirms that angiotensin receptor blockers and angiotensin converting enzyme inhibitors can improve insulin resistance (76). One study showed that lisinopril increased insulin sensitivity and reduced total and free testosterone in PCOS patients with hypertension (77). Angiotensin receptor antagonists and angiotensin converting enzyme inhibitors have positive effects on symptom improvement in PCOS patients. As mentioned above, both hyperandrogenism and insulin resistance are improved by hindering the RAS system, which may provide a new strategy for treating PCOS (77).

Additionally, some research have shown that the D allele of the ACE gene may be linked to acanthosis and a greater risk of insulin resistance in PCOS patients (78, 79). Other investigations have verified that, despite the ACE gene polymorphism not being linked to the pathophysiology of PCOS, it may be strongly linked to a number of metabolic diseases such insulin resistance, hyperlipidemia, and hyperandrogenemia (80, 81). And because it is bound up with steroid production in the ovary, it may also be connected with the exacerbation of clinical expressions of PCOS (82).

## Ovarian cancer

Ovarian cancer (OC) is the most deadly kind of cancer that may develop in a woman’s reproductive system. OC seriously affects life and health, but its pathogenesis remains unclear at present. The formation of tumors may be linked to the enhanced local expression of Ang II and its receptors in the ovary (83), Ang- (1-7), on the other hand, can reduce the growth, migration potential and invasiveness of tumors by several mechanisms against Ang II.

Takayasu et al. (84) Studies examining AT1R expression in human ovarian cancer and attempting to determine whether AT1R blockers could inhibit tumor progression showed AT1R expression in 85% invasive ovarian adenocarcinomas, 66% borderline malignancies, and 14% benign cystadenomas. In AT1R positive patients, there was a considerable increase in the amount of VEGF expression and tumor microvessel density. *In-vitro* studies proved that AT1R blockers could lessen the invasion potential and VEGF secretion of ovarian cancer cells. Previous research has shown that invasive and borderline diseased epithelial ovarian tumors had greater AT1R levels than healthy ovaries. Furthermore, earlier research has shown that individuals with ovarian cancer who have high AT1R levels had a poorer prognosis (83). Serum ACE levels are elevated in OC patients. As for epithelial OC patients, serum ACE levels do not vary according to patient stage and pathological subtype. This situation suggests that serum angiotensin I converting enzyme may be a marker of disseminated germinoma (85).

Ang II and ovarian cancer cells can hasten the metastasis of epithelial ovarian cancer (EOC) through interaction (86). Higher blood AT1-AA titers were shown to be related with the late advancement of EOC in patients, according to the findings of another investigation. What’s more, higher serum AT1-AA titers may significantly contribute to the progression of EOC by facilitating cancer cell migration and angiogenesis (87). In ovarian cancer, high levels of AT1R expression are linked to increased rates of tumor angiogenesis and a poor prognosis, and AT1R blockers have been shown to inhibit peritoneal spread of ovarian cancer in a mouse model (88). *In vitro* and *in vivo* experiments on OC cell lines have shown that Candesartan, an AT1 receptor antagonist, can not only inhibit the invasion ability of tumor cells *in vitro*, but also inhibit the growth and progression of tumor and the formation of blood vessels in mice (84, 88). Therefore, a series of studies have confirmed that Ang II and Ang- (1-7) can participate in the pathological process of ovarian cancer by affecting the proliferation, migration and angiogenesis of tumor cells (6, 55, 89, 90).

## Endometriosis

Endometriosis (Ems) is an estrogen-dependent condition marked by the development of adenocarcinoma and endometrial stromal cells outside of the uterine covered mucosa and myometrium. Although endometriosis is a benign disease, it can result in abdominal pain, menstrual abnormalities, and infertility, which disadvantageously affects about 10 percent of women in childbearing age (91).

The pathogenesis of endometriosis remains unclear. Worse still, the etiology and pathology of infertility are more complex. In the endometrium of people with endometriosis, there is enhanced expression of the Ang-1-7 receptor Mas, and the high expression of Mas may further the occurrence of endometriosis (92). Angiotensin type 1 receptor is involved in angiogenesis and affects endometrial growth, invasion and regression (93). In another trial using the AT1R antagonist telmisartan to treat mice endometriosis, it inhibited angiogenesis, immune cell content, and lesion growth in endometriosis (94). Tanshinone IIA not only significantly inhibits the growth of ectopic endometrium, but also alleviates EMs-induced pain syndrome by regulating RAS in neurons (95). However, on account of the physiological differences between human and mouse endometrium, a more complete theoretical and experimental basis is essential for applying this result to patients with endometriosis needs.

Studies on endometriosis and ACE gene polymorphism suggest that ACE gene polymorphism has some connection with the susceptibility and development of endometriosis. Furthermore, the ACE gene polymorphism can inhibit EMS-related pain by blocking the expression of Ang II and AT2R (95–98). On the other hand, some studies have borne out that ACE gene polymorphisms and haplotypes are not associated with endometriosis, and no association has been found between ACE gene polymorphisms and the prevalence, progression, or number of lesions of endometriosis (99–101).

## Endometrial cancer

Endometrial cancer (EC) is a primary epithelial malignant tumor of the endometrium, accounting for 20%-30% of female reproductive tract malignant tumors. It is generally believed that abnormal hormone secretion, genetic factors and environmental factors are connected to the onset and progression of the illness (102).

As we all know, the endometrium is responsible for the expression of all RAS components. The mRNA levels of AT1R, ACE1, and ACE2 were shown to be greater in tumor tissues of patients with endometrial cancer (103), compared to the levels found in neighboring non-cancerous tissues. The high expression of these factors supports the possibility that RAS contributes significantly to the emergence of endometrial cancer. The impact of Ang II on endometrial cancer cells was investigated in a study using endometrial cell lines. The results indicated that Ang II could augment the potential of cancer cell proliferation, metastasis and adhesion, and was associated with the highly differentiated state of cancer cells (104). However, to some degree, the capacity of tumor cells to migrate and invade may be impeded by silencing of the Ang II AT1R receptor, but this change is not absolute (105). Hence, Ang II may have a more complex network of effects in endometrial cancer, which needs further exploration. In endometrial cancer, Ang II increases the production of VEGF in a dose-dependent way and stimulate angiogenesis. Overexpression of Ang II-degrading enzymes and small interfering RNA were able to suppress both the immunoreactivity of VEGF as well as the number of blood vessels that were present in the tumor (106). One study observed that the AT1R blocker telmisartan significantly inhibited the growth of human endometrial tumors without toxic effects. This may provide a new strategy for future treatment of EC (105).

ACE gene polymorphism may be associated with the pathogenesis of endometrial cancer, and may be related to the development of the disease as well as the age of onset (31, 107, 108). Ang II may affect the progression of endometrial cancer by promoting the proliferation, differentiation and invasion of tumor cells. At present, few data can throw light upon the expression of Ang- (1-7) and its receptor in endometrial cancer and its effect on endometrial cancer, which may be the direction of future research, so as to further improve the research in endometrial cancer.

## Preeclampsia

Preeclampsia (PE), which is a distinct pregnancy illness and a leading cause of maternal, fetal, and neonatal mortality with no proven cure other than delivery, is defined as the start of new-onset hypertension and proteinuria after 20 weeks of gestation (109). According to the performance of different pregnancy stages, PE is divided into early onset and late onset. Although the symptoms are similar, the causes of the two types are not the same (110). Early-onset PE is more commonly thought to be due to defects in placental development, so we are going to put our attention on early-onset PE.

Although the specific pathogenesis of PE is not adequately understood, numerous studies have shown that placental ischemia caused by reduced uteroplacental blood flow is the primary foundation of this pregnancy condition (111, 112). In PE pregnant women, the levels of Ang II and Ang- (1-7) were found to be much lower than those seen in normal pregnant women. Interestingly, the pressor response of Ang II and the amount of autoantibodies to the AT1 receptor are elevated (113). At1-aa can give rise to vasoconstriction through AT1R on vascular smooth muscle, and also promote the upregulation of various active substances (114–116), among which NADP upregulation enhances ROS formation, PAI-1(plasminogen activator inhibitor -1) reduces trophoblast invasion (117, 118). The increase of circulating sFlt1 level can result in the decrease of VEGF and PLGF bioavailability (50). Clinically, hypertensive individuals’ risk of developing PE may be determined by assessing the ratio of circulating sFlt1/PIGF, which has the highest sensitivity and specificity in predicting and diagnosing PE. The decrease of free VEGF level can induce the expression of endothelin-1, touch off the decrease of renin secretion, further strengthen the negative regulation of blood volume, and thus cause the poor perfusion of placenta, which are associated with the development of the illness in its various stages (119). Aside from this, the increase of placental renin gene expression and protein level in transcription factor replacement and (Stox1) knockout mice gave rise to gestational hypertension through this change, which was alleviated by Ang II receptor blocker treatment, indicating that stox1 contributes to the development of PE through Ang II (120, 121).

There are different opinions on the expression of Ang- (1-7) in PE. One research revealed that early in pregnancy, PE patients’ levels of Ang-(1-7) were greater than those of normal pregnant women, and this difference only existed in women with female fetuses (122). We believe that the pathogenesis of PE has a bearing on the imbalance of RAS. In future studies, we need to learn more about how Ang II and Ang- (1-7) are expressed and look into the reasons why PE patients have atypical Ang II and Ang- (1-7) expressions. Patients with PE have RAS disorder, especially the activation of AT1R can participate in the pathogenesis of PE by promoting the validation reaction-mediated oxidative stress and other pathways (123). Angiotensin antagonists and angiotensin-converting enzyme inhibitors can lower blood pressure, but cannot be recommended as oral drugs due to their teratogenic effects.

## Coronavirus disease 2019

Coronavirus disease 2019 (COVID-19) is an infectious disease caused by severe acute respiratory syndrome coronavirus 2 (SARS-CoV-2). It is associated with respiratory tract involvement and typical symptoms include dry cough, dyspnoea, fever, etc (124). In addition, the understanding of whether women’s reproductive systems can be affected by the coronavirus continues to improve (125). ACE2 is a key enzyme in the RAS of this disease, not only regulating the expression of Ang II and Ang-(1-7), but also acting as a receptor to mediate infection of target cells by SARS-CoV-2 (126). ACE2 is present at all stages of follicular development. Ang II is mainly expressed in granulosa cells and Ang-(1-7) is expressed primarily in intermembrane cells. Both Ang II and Ang-(1-7) are involved in the development and maturation of oocytes (6). Two studies in women of childbearing age showed no significant effect of SARS-CoV-2 on ovarian reserve function (127, 128). Another analysis of 32 in vitro fertilization (IVF) patients revealed there were no obvious differences in follicular function, including but not limited to hormone synthesis, trigger response and embryo quality (129). However, it was also suggested that SARS-CoV-2 could cause a decrease in ACE2 expression, leading to an increase in Ang II and an imbalance in the expression of Ang II and Ang-(1-7) in the ovary. Consequently, it induced enhanced local inflammation, fibrosis, apoptosis and other pathological responses in the ovary, further affecting the reproductive function of the ovary. The most attention in research was paid to the effect of SARS-CoV-2 on menstruations. A series of studies indicated that SARS-CoV-2 has a significant impact on menstrual patterns, menstrual volume and dysmenorrhea (4, 130, 131). This might be because ACE2, Ang II and Ang-(1-7) can maintain cyclic changes in the endometrium by participating in vasoconstriction as well as cell renewal and proliferation. Since ACE2, which functioned as the SARS-CoV-2 receptor, was low in abundance in the endometrium, it did not show susceptibility to the coronavirus (132). Intriguingly, studies suggested that female age can be a risk factor for COVID-19. This was achieved because the expression of ACE2 in the endometrium increased with age (4). ACE2 was widely expressed in the placenta, including trophoblast cells, perivascular and vascular smooth muscle cells of decidual vessels as well as decidual stromal cells (133). This inferred that the placenta was more prone to the effects of SARS-CoV-2. Furthermore, the expression of ACE2 was not constant in the placenta. The level of ACE2 expression decreased with increasing gestational age, suggesting a greater likelihood of transplacental transmission of SARS-CoV-2 in early gestation (134). Although it was theoretically possible for SARS-CoV-2 to be transmitted to infants through the placenta. However, studies demonstrated that most newborns were not found to be infected after birth. It was hypothesized that this was possibly due to the barrier effect of the placenta, but the exact mechanisms of action remained unclear (135). Other than the possible effects mentioned above, SARS-CoV-2 could also affect endocrine organs such as the thyroid gland, thus impacting the endocrine system of patients (136, 137). This might further cause alterations in female fertility. Due to limited clinical data, the understanding of the impact of SARS-CoV-2 on the female reproductive system is not comprehensive and profound. Therefore, predicting the impacts of SARS-CoV-2 on the female reproductive system and the specific mechanisms of action will be an essential direction for future research.

## Conclusion

In conclusion, the female reproductive system does in fact include a local renin-angiotensin system. Numerous studies have also demonstrated that angiotensin, including Ang II and Ang- (1-7), can be produced locally in the reproductive system. RAS affects oocyte maturation and excretion, endometrial periodic changes and hormone production. RAS is considered to be an important way to regulate physiological functions of the reproductive system. However, the functions that Ang II and Ang- (1-7) play in the physiology and pathology of the female reproductive system are still not fully understood. Furthermore, most of the previous studies remain at the level of nonfunctional description. The intricate dependency network in the peptide-hormone system in issue, together with species variations, are the main causes of the interpretation challenges. Undoubtedly, further study in this field is required to fully comprehend how RAS affects female fertility. Likewise, the intensive study in the future is indisputably essential to determine the contribution of RAS to a wide range of related diseases.

## Author contributions

YL and ZZ contributed to conceived and designed the review. YL wrote the paper. RJ and HH did the document retrieval. LZ and MC polished the paper. ZZ and WP checked the paper. All authors listed have made a substantial and intellectual contribution to the review and approved it for publication.
